# Assessment of Smart Watches for Management of Non-Communicable Diseases in the Ageing Population: A Systematic Review

**DOI:** 10.3390/geriatrics3030056

**Published:** 2018-08-29

**Authors:** Lachlan A. N. Gordon

**Affiliations:** 1 School of Medicine, University of Queensland, Brisbane 4006, Australia; lachlan.gordon@gmail.com; Tel.: +61-(2)-6244-2222; 2 Canberra Hospital, Garran 2605, Australia

**Keywords:** elderly, wearable technology, falls, telemedicine, eHealth

## Abstract

Advancement in wearable technologies is providing promising new ways to monitor and improve patient care to the ageing population. With the global demographic transition of developed countries to an ageing population, implementation of these technologies could benefit patients and clinicians. This systematic review assesses experimental studies performed utilizing these technologies. A systematic review of peer-reviewed literature was performed on the application of wearable technologies in the patients 60 years old or greater or what is considered ageing population. Search results were reviewed and synthesized to attempt to ascertain its possible clinical application and impact. A total of 422 papers were identified for review. Eight papers were relevant to the ageing population. The majority of papers identified were experimental studies. This was because the technology is still new to the field of medicine. The studies were performed in North America, United Kingdom, Germany and Indonesia. All showed promise that wearable technologies can benefit the management of non-communicable diseases in the ageing population. Current studies focus on the experimental nature of wearable technology. Further clinical trials are needed to assess the benefit in the management of ageing populations in the clinical setting.

## 1. Introduction

The ageing population in developed nations is growing. Better technologies, research and access to modern medicine contribute. Elderly people within society are more at risk of non-communicable diseases, for example cancer, cognitive decline, falls, cardiac diseases, diabetes and respiratory diseases [1]. Managing these conditions may be labor intensive, require expensive investigations and management as well as admissions to care facilities. However, with the advancement of technologies and wearable devices, a new area of preventative, monitoring and early warning for medicine has been developed [2]. More populations are implementing wearable devices for improvement in fitness, health and communication. Major technology companies have also realized the potential of these devices in healthcare. The progression from personal alarms to “smart watches” enables changes in the management of non-communicable diseases.

In Australia, there are approximately 3.7 million Australians aged 65 and over. Seventy-one percent had self-reported data of stroke, 59% cardiovascular disease, 30% experienced at least one fall per year and 8.8% have some form of dementia. This population contributed to 20% of all presentations to emergency departments from 2014–2015. The main three diagnoses were recorded as: (1) “Pain in throat and chest”, (2) “abdominal and pelvic pain” or (3) “syncope and collapse”. All of these conditions could be monitored, assist in rapid access to emergency ambulance services and potentially avoided with the use of wearable technologies [3].

A systematic review of the literature was performed to assess the healthcare applications in the ageing population and the possibility of use as a personal alarm and a monitoring tool for patients’ health and well-being in the community.

## 2. Methods

An initial review of appropriate medical and technology databases was performed. Five databases were subsequently identified to enable the performance of a systematic review of the literature regarding wearable technologies and health care in the ageing population.

### 2.1. Search Terms

**Figure A1:**
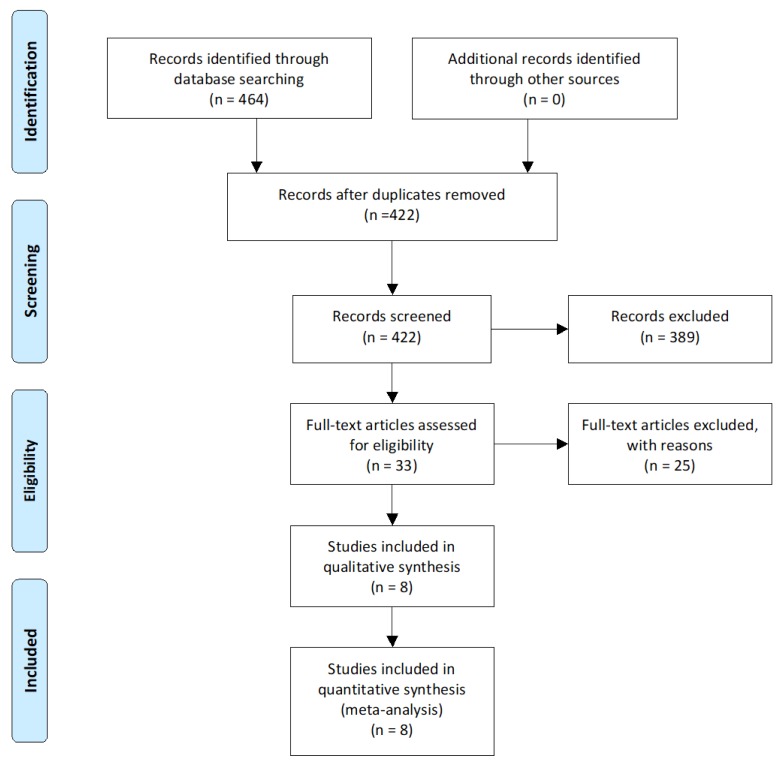
Systematic review summary table [14].

A systematic review of published literature following the PRISMA (Figure A1) guidelines was performed utilizing the five databases identified. Search terms were developed utilizing key words identified from various international sources. The search terms included were “ageing population”, “elderly”, “wearable technology”, “wearable device”, “smart watch” and “healthcare”. Exposure terms used included “falls prevention”, “aged care”, “healthcare monitoring”, “improved outcomes” and “non-communicable diseases”.

### 2.2. Information Sources

Databases most appropriate for the literature search included PubMed, EMBASE, IEEE Xplore the Cochrane Library and MedlinePlus.

- PubMed, is a database of life science and biomedical literature
- EMBASE, is a database of biomedical and pharmacological literature
- IEEE Xplore, is a database of computer science, electrical engineering and electronics and allied fields
- The Cochrane Library, is a collection of databases and systematic reviews in medicine and other healthcare specialties
- MedlinePlus, is a database that is produced by the United States National Library of Medicine

As well as database searches, key references and prominent authors were identified resulting in a well-rounded complete assessment of the literature available.

### 2.3. Eligibility Criteria

As research on wearable technologies is limited before 2004. Articles reviewed were published from 2004–2018. Inclusion criteria included all publications written in English or with an English translation. All publications with a clear link to wearable technologies, healthcare and ageing population were included. Publications were assessed on their study designs and methods and there were no exclusions. Publications related to specific non-communicable disease monitoring included falls, stroke, dementia and cardiovascular risk factors. Studies excluded were assessing the usability of the technology for older populations and not referring to a specific healthcare risk factor application. Non-research publications and opinion articles were excluded. Grey literature was not included in the systematic search. However, global publications from the World Health Organization (WHO), Centers for Disease Control (CDC) and national government organizations including the Australian Institute of Health and Welfare (AIHW) were included and discussed in the review. Similarly, information regarding specific wearable technologies and “smart watches” was accessed from technology websites to assist in providing context and discussion. 

### 2.4. Study Selection

Research papers were identified through a screening process executed by a single author. Initially articles were screened by relevance base on the paper’s title. Subsequently a review of the abstracts was performed to assess relevance to the topic of wearable technologies in healthcare and the ageing population in relation to non-communicable diseases. Finally, the strength of the study designs was assessed. However, as wearable technology in healthcare is a new concept this was not a complete limiting factor.

### 2.5. Risk of Bias and Quality Assessment

As wearable technology in healthcare is a relatively new concept there was a limited number of studies to assess. Randomized control trials on this subject are difficult to perform and were neither expected during the literature search nor found. As the author is an advocate for technology application in the healthcare setting, some subjective selection bias may be present. However, risk of bias was assessed using the Systematic Review Critical Appraisal Skills Program (CASP) checklist. This revealed that the review question was addressed, and relevant studies were included.

### 2.6. Summary Measures

Papers identified were summarized in relation to study design, setting, exposure, outcome, key findings and statistics when available. An assessment of the study, its impact and the potential for implementation of wearable technologies was also performed in relation to the conclusion of the literature reviewed.

### 2.7. Study Assessment

The papers were assessed, and study designs were compared and contrasted. Discussion was developed in relation to the approaches of the studies and the assessment of wearable technologies in healthcare and the elderly populations. Studies were assessed on the impact and potential for implementation of wearable technologies. The current evidence was assessed in relation to the possible improvement of outcomes in the social, economic and cultural context. Specific attention to the context of globalization and development in the healthcare setting will also aid discussion of impact of these technologies in the ageing population. Discussion on future applications and research into wearable technologies in managing health care problems in the ageing population will also follow.

## 3. Results

The search identified 3611 papers for review. Refining the search terms reduced the papers to 422. PubMed provided 338, IEEE Xplore 83, Cochrane Library 1, Medline 0 and EMBASE 0. Duplicates were identified and removed. Thirty-three papers were identified as pertaining to an aged population and healthcare setting. Subsequently 8 were identified for the systematic review.

Identified papers for review assessed wearable technologies in elderly patients for the management of medication compliance (*n* = 2), falls (*n* = 4), longitudinal health monitoring (*n* = 1) and monitoring for neurological disorders (*n* = 1). 

### 3.1. Study Settings

Studies were performed in North America [4,5,6,7], United Kingdom [8,9], Germany [10] and Indonesia [11]. The two studies that assessed elderly patients’ compliance with medication were performed in Germany [10], and the United States of America (US) [6]. The Neurological Disorders study by Saunders and Clements was performed at the Georgia Institute of Technology in the United States [7]. The studies related to falls were performed internationally. No studies related to wearable technologies in elderly patients were identified in Australian literature. However, given the international nature of studies identified and the similarity of the elderly demographics, this data could be extrapolated to the Australian population or any ageing population in stage 4 of the demographic transition.

### 3.2. Study Designs

The four studies assessing smart sensors and wearable technologies in the settings of falls in the elderly populations were designed to be experimental studies performed in a controlled environment [4,8,9,11]. They compared the outcomes of a technology sensing that a patient had a fall with the overall health outcome for the patient. The two studies assessing medication compliance were prospective cohort studies [6,10]. The Neurological Disorders study was designed to assess bradykinesia, tremor and postural instability of symptoms associated with Parkinson’s Disease [7]. They implemented a prospective cohort study to assess that. Finally, the assessment of longitudinal health monitoring was a prospective study of a single subject with assessment of physiological measurements associated with measurements recorded by the smart watch [5]. All studies utilized various statistical analyses to assess their results.

## 4. Themes

### 4.1. Use of Wearable Technology in Fall Detection and Prevention

All studies were assessing the utilization of wearable technology in the setting of a falls detection system. The study performed by Casilari and Oviedo-Jimenez simulated experiments utilizing a “smart watch” and a “smart phone” to detect falls in “patients” then call for help. The sensitivity for a type of fall forwards, backwards or lateral was measured to be between 80% and 100% accurate with specificity of 90%. This suggested that the utilization of wearable technologies may assist in the detection of falls. However, the occurrence of “false alarms” may reduce the effectiveness of this technology. The false alarms were triggered by the various mobility modes of the patients [8].

Rakhman et al. took the technology one step further to utilize a smart phone as a wearable device to monitor falls and signal an alert to another person. The simulated experiment showed that there was an 86.67–96.67% true positive rate as to the direction of fall. The fall backward had the highest false negative rate at 13.33%, where as a fall forward had the lowest at 3.33%. They also attempted to recognize the activity of daily living prior to the fall and were able to accurately identify that between 90% and 100% of the time [11].

Sixsmith and Johnson performed experimental studies on wearable sensors to detect falls. The initial results of these trials were not encouraging and indicated that the alarm wasn’t triggered for almost 66% of falls. They postulated that this might have been improved if the sensitivity was higher. However, with higher sensitivity there may have been a higher false positive rate [9].

Chaudhuri et al. assessed the real-world accuracy and use of a wearable fall detection device. They measured 42.2% false positive rate and partial completers had 25 false alarms. Over 263 days (9.5%) (*p* = 0.31). The study showed the critical need for real-world testing of fall devices in an attempt to improve the accuracy of this technology [4].

### 4.2. Use of Wearable Technology in Medication Adherence

Sailer et al. performed a proof of concept experimental study with the view to develop a randomized study in the future. Their results showed that other than just reminding the patient to take their medications there was the ability to visualize the types of drugs they needed to take. They postulated that would lead to increased compliance among users [10].

Kalantarian et al. postulated a method of recognizing wrist gestures to indicate that the patient had taken their medication as another measure of medication compliance. Unfortunately, their results showed a very high false-positive rate of pill cap opening detection. The main effect confusing results was related to similar motions of bottle opening. A survey of 221 individuals was performed to validate the motion detection designed to identify how to improve the accuracy of the device. Their results showed that individuals would need to adapt their “smart watch” to recognize the method of pill cap opening suggested in their paper [6].

### 4.3. Use of Wearable Technology in Assessment of Neurological Disorders

Saunders and Clements documented the performance of wearable technologies for assessing symptoms in Parkinson’s Disease (PD). The results showed statistically significant features for the sit-to-stand and stand-to-sit (STS) and the X (vertical) positive jerk score (*p* < 0.001). When data was sorted by activity type there was evidence that there was normal verses PD discrimination. Symptoms in the setting of rest, STS and walking with turns (WWT) were also assessed. The combination of gyroscope and accelerometer features allowed for 95% correct classification in to the correct category of activity compared with 75% for accelerometer measurements alone. Low statistical significance was identified when assessing tremor classification. This suggested that with the use of wearable accelerometers and gyroscopes Parkinsonism movements could be identified in individuals [7].

### 4.4. Use of Wearable Technology in Longitudinal Health Monitoring

Jovanov assessed the continuous physiological monitoring of the heart rate by a smart watch over a continuous period of 120 days. They concluded that only 11.12% of records were missing during that time and the majority of that can be attributed to watch charging time. Some data was lost because of poor contact or motion artifacts. They suggested that wrists were not very good locations for physiological sensors as there are motion artifacts and access to the skin provides less reliable measurements. They did not assess a quantifiable health outcome but recognized the potential for continuous physiological monitoring [5].

## 5. Discussion

In developed countries the demographic and epidemiological transition is progressing to an ageing population with the development of significant non-communicable disease. In the elderly population this consists of a significant proportion of chronic diseases including cardiovascular, neurological and respiratory problems. Advancements in wearable technologies may be able to assist the elderly populations in monitoring of these conditions. Because these technologies are very new, limited studies have been published assessing these applications.

The specific application of falls detection and alarms requires further research until technologies can be used in a clinical setting. The high false positive rates amongst all experimental studies reviewed suggest that further studies need to be performed to improve the accuracy of these applications prior to implementation in a clinical environment. It is clearly a pertinent issue in the elderly population with 30% of that population experiencing falls in Australia [3]. The early detection of the these falls in the clinical and home environment is pertinent to the patient outcomes. All studies recognized the importance to perform further studies and to improve the accuracy of these technologies. They have the potential to decrease morbidity to the patient. It is clearly difficult to perform randomized trials on new technology when not completely validated in the experimental setting. However, the implications of a technology are significant for the improvement of elderly patient outcomes following falls.

Utilizing a smart watch to improve medication compliance in the elderly population shows promise. However, this is a costly alternative to the currently well-established practice of Webster packs [12]. Although despite that, it could be implemented in the setting of a prompt to inform patients to take their medications at the correct time. This would avoid patients forgetting to take their medications. The application of measuring when the patient opens the bottle to take their medications showed very little clinical application. That is because there is no real method of detecting that the patient took the correct medication or was even taking a medication at the time of the algorithm measuring the gesture. Despite all that, it cannot be understated that the clinical application of subtle reminders to take regular medication would improve medication compliance across all age groups and could be particularly useful in the setting of the ageing population in the situation of cognitive impairment [13].

Measuring neurological signs in the setting of Parkinsonism tremors in elderly patients could be a promising application for the early detection of these neurological disorders. This could lead to earlier treatment and better management of these conditions in the future.

The application for longitudinal physiological monitoring shows promise with many devices on the market presently that can perform this task. The use of these in monitoring in real time in the clinical setting is invaluable. Current large cumbersome monitors that rely on electro-cardiograph leads, for example decrease patient mobility and can be quite limiting. The use of a “smart watch” to do this could be easier. The application of long-term heart rate monitoring of Jovanov showed that over a continuous period of 120 days there was only 11.12% of lost data and that this was mostly attributed to device charging [5]. Because of this they recognized that the potential for continuous physiological monitoring in patients showed promise. However, for this application to be utilized in the clinical setting more research and development of the technology is necessary.

Despite all these experimental applications of wearable technologies to improve various clinical outcomes for the ageing population, further research is required. This would include clinical trials to ascertain whether or not a smart watch could truly have a significant impact on various management aspects of the elderly population.

## 6. Limitations

There are significant limitations within the studies reviewed. All studies were early experimental studies to ascertain all the possible clinical applications of wearable technologies in the elderly. Formal clinical trials would be needed to prove if these technologies would truly work clinically. However, despite this it would be difficult to performed randomized trials in this setting.

## 7. Recommendations for Future Research

Wearable technology is an evolving area for the field of healthcare with the majority of applications targeted at fitness. The difficulty would be that establishment of formal clinical trials may pose certain ethical dilemmas particularly in the setting of elderly patients. Recommendations for any research in to the validity and benefit of wearable technology in healthcare setting should encompass assessment of measured application, patient acceptance and usability as well as practical functionality.

## 8. Conclusions

The use of wearable technologies for the assistance in the management of non-communicable diseases looks promising as further technology and research is performed and developed. With a global transition to utilizing these technologies for health, fitness and well-being the devices show promise that they could provide benefits for patients and clinicians.
